# An objective function for full-waveform inversion based on frequency-dependent offset-preconditioning

**DOI:** 10.1371/journal.pone.0240999

**Published:** 2020-10-28

**Authors:** Sérgio Luiz E. F. da Silva, Pedro T. C. Carvalho, Carlos A. N. da Costa, João M. de Araújo, Gilberto Corso

**Affiliations:** 1 Departamento de Física Teórica e Experimental, Universidade Federal do Rio Grande do Norte, Natal, RN, Brazil; 2 Departamento de Biofísica e Farmacologia, Universidade Federal do Rio Grande do Norte, Natal, RN, Brazil; Universidade Estadual de Maringa, BRAZIL

## Abstract

Full-waveform inversion (FWI) is a powerful technique to obtain high-resolution subsurface models, from seismic data. However, FWI is an ill-posed problem, which means that the solution is not unique, and therefore the expert use of the information is required to mitigate the FWI ill-posedness, especially when wide-aperture seismic acquisitions are considered. In this way, we investigate the multiscale frequency-domain FWI by using a weighting operator according to the distances between each source-receiver pair. In this work, we propose a weighting operator that acts on the data misfit as preconditioning of the objective function that depends on the source-receiver distance (offset) and the frequency used during the inversion. The proposed operator emphasizes information from long offsets, especially at low frequencies, and as a consequence improves the update of deep geological structures. To demonstrate the effectiveness of our proposal, we perform numerical simulations on 2D acoustic Marmousi2 case study, which is widely used in seismic imaging tests, considering three different scenarios. In the first two ones, we have used an acquisition geometry with a maximum offset of 4 and 8 km, respectively. In the last one, we have considered all-offsets. The results show that our proposal outperforms similar strategies, for all scenarios, providing more reliable quantitative subsurface models. In fact, our inversion result has the lowest error and the highest similarity to the true model than similar approaches.

## Introduction

An important task at exploration geophysics is the seismic imaging of underground structures such as gas and oil deposits [1, 2]. The main focus of seismic imaging methods is to produce maps of the Earth’s subsurface from recorded data analyses. Several imaging seismic methodologies have been employed in the industry such as refraction tomography [3] and seismic travel-time tomography [4, 5]. Nowadays, a technique that has received a lot of attention is based on the manipulation of the complete waveform and is called full-waveform inversion (FWI) [6, 7]. Originally developed within seismology [8, 9], FWI has successful applications in exploration seismic (e.g. [10, 11]) which has attracted interest in many other areas such as applications in ultrasonic medical tomography [12], solar interior studies [13] and ground-penetrating radar [14]. The strong point of the FWI is to exploit the full information of waveforms to simulate all phenomena wave, whilst most of the imaging techniques use only the travel-time kinematics information.

From a practical viewpoint, FWI is a data-fitting technique that aims to infer the physical parameters of geological structures using the wave equation solution [6, 7, 15]. FWI is an interesting inverse problem in which the inverse problem consists of obtaining the model parameters, which is data-driven by using an optimization algorithm, usually a gradient-based method [7]. The forward problem consists of simulating the wave propagation from a seismic source to the receivers using the wave equation. Typically, in the optimization process, the objective function is based on the misfit between modeled and observed seismic data, in which the optimal solution is given by the global minimum of this function [6]. However, from a practical viewpoint, the global minimum is utopian for the FWI problem. Thus, several techniques of preconditioning of the objective function have been proposed to determine a local minimum that is close to the global minimum [7, 16, 17].

Despite its great potentiality, the FWI is an ill-posed problem in the sense of Hadamard [18], which means that the solution is not unique. From an optimization viewpoint, the inversion process can be trapped in a local minimum [6], and therefore the results may be unsatisfactory. A commonly used strategy to reduce the FWI ill-posedness is based on a multiscale approach [19, 20], in which the inversion is carried out sequentially by considering, firstly, the low-frequency-data components and then the high-frequencies components. In this way, we need to adopt efficient strategies, such as the multiscale approach aforementioned, as well as incorporate as much information as possible in the solution of the inversion problem to steer the inversion towards a reliable seismic image.

In general, all seismic traces provide the same contribution to the inversion process independently of other seismic acquisition parameters. However, the wise use of information from seismograms is crucial to obtain a reliable subsurface model. Thus, several workflows have been proposed to manage the information during the inversion process such as inversion from primary reflection [21] and a combination of refraction and reflection FWI (e.g. Ref [22]). Reference [23] (pp.193-197) proposed an inversion strategy based on a smart selection of receivers, in which a trace grouping for each iteration is chosen, and the inversion is performed by alternating groups with less information and, at each iteration, the amount of seismic traces to be used in the inversion process is increased. Recently, inspired by the fish sensory system, Ref [24] proposed a processing methodology by alternating the groups of traces according to the signal frequency. In a comparable research line, Ref [25] proposed a weighting operator based on the source-receiver-distance (offset) to diminish the contribution of the high-amplitude direct water wave along the inversion process as an automatic way to select information from receivers during the FWI processing.

Nowadays, the new acquisitions geometries allow us to record waveforms with an angular aperture (wider offsets) much larger than a conventional survey [26], which is very important to better illuminating the deeper regions of the medium under study. They also allow us to evaluate the lower frequencies of the subsurface model [27]. Despite these new acquisitions to be efficient to illuminate the deeper part of the subsurface model, due to the wide-angle aperture that they offer, the waveforms’ information recorded by large-offsets should be included in the inversion process with caution. This is since if we start the inversion from a reasonable initial model, the travel-time error is large to wide-offsets. In other words, the waveforms recorded in the largest offsets are the most non-linear components of the seismic data [28].

In this work, we consider a frequency-domain acoustic FWI scheme that combines the ideas from the multiscale inversion strategy [19, 20] with an improvement from the weighting operator introduced by Ref [25]. We propose a frequency-dependent offset-weighting operator in order to precondition the data according to signal frequency and source-receiver distance (offset). In this perspective, the weighting operator emphasizes the contribution of long offsets which are rich in large wavelengths waveforms information (low-frequency information), which generally carry much information on the deep structure that is dominated by diving waves. In fact, diving waves propagate in a great angular range, and therefore, it is carrying important information from the deep region of the subsurface. Simultaneously, the proposed operator decreases the contribution of the direct wave and other waves running through shallow underground structures, which are benefited by data redundancy.

This paper is organized as follows: First, in Section Methodology, we give a brief overview of FWI formulation, in which we present in some detail the design of the weighting operator, which is our original contribution. Then, in Section Numerical Example, numerical experiments on a 2-D acoustic frequency-domain full-waveform inversion are presented, and the main results are illustrated by using the Marmousi2 model [29]. In these experiments, we consider three different scenarios in sense of maximum offset: (i) 4km; (ii) 8km, and (iii) all receivers are considered (all-offsets). To conclude, in Section Conclusion, we discuss the gain of our strategy and its potential implications.

## Methodology

### Conventional FWI approach

FWI is conventionally formulated as a constrained least-squares optimization problem [6], in which it can be expressed in the frequency-domain as:

$$\begin{array}{c} \underset{m,u}{min}\;\frac{1}{2}\sum_{\omega }\sum_{s,r}\|\Gamma_{s,r}u_{s}\left(\omega \right)-d_{s,r}\left(\omega \right){\|}_{W_{s,r}}^{2}\;subject\;to\;A\left(m,\omega \right)u_{s}\left(\omega \right)=q_{s}\left(\omega \right), \end{array}$$ (1)

in which *m* is the model parameters, *ω* represents the angular frequency, *u*_*s*_ is the acoustic seismic wavefield generated by source *s*, Γ_*s*,*r*_ represents the sampling operator (onto the receiver *r* of the shot *s*), *d*_*s*,*r*_ is the observed seismic data. $\|Z{\|}_{W_{s,r}}^{2}=Z^{†}W_{s,r}Z$ represents the *l*_2_-norm squared with weighting operator *W*_*s*,*r*_ applied to the residual data. The constraint in (1) represents the wave equation in a compact form, in which *A*(*m*, *ω*) is the Helmholtz operator (or impedance matrix) [30, 31] and *q*_*s*_ represents the seismic source. Furthermore, this constraint is a linear system equation [30] for each angular frequency and, its solution is the wavefield corresponding to the whole model. However, in practice, we only have wavefield information at the receiver positions. Thus, the sampling process is necessary by using the Γ operator to compare the observed data with the simulated data.

In this work, we consider the frequency-domain wave equation, for an acoustic medium with velocity *c*, which can be written as:

$$\begin{array}{c} \nabla^{2}u\left(x,\omega \right)+\frac{\omega^{2}}{c^{2}\left(x\right)}u\left(x,\omega \right)=f\left(\omega \right)\delta \left(x-x_{s}\right), \end{array}$$ (2)

in which ∇^2^ is the Laplacian operator, *u*(**x**, *ω*) the modeled acoustic seismic wavefield in the frequency-domain at the spatial location **x**, and *f*(*ω*)*δ*(**x** − **x**_*s*_) represents the frequency-domain source at the position **x** = **x**_*s*_ for *δ* the Dirac Delta function. Comparing the constraint in Eqs (1) and (2), we have that the impedance matrix is: *A*(*m*, *ω*) = *L* + *ω*^2^ *diag*(*m*), with *L* the discretized Laplacian operator and the parameter *m* is the squared slowness: *m* = 1/*c*^2^(**x**). It is important to point out that, the spatial coordinate (**x**) is implicit in Eq (1) and, henceforth for the sake of a simplified notation.

An elegant approach to solve the problem formulated in (1) can be by introducing an augmented Lagrangian functional [32]

$$\begin{array}{c} L\left(m,u_{s},v_{s}\right)=\frac{1}{2}\sum_{s,r}\|\Gamma_{s,r}u_{s}-d_{s,r}{\|}_{W_{s,r}}^{2}+\sum_{s}{\langle v_{s},A(m)u_{s}-q_{s}\rangle }_{x} \end{array}$$ (3)

where 〈.〉_**x**_ is the inner product on the spatial coordinates **x** and *v* is the Lagrange multiplier. The angular frequency (*ω*) is implicit in Eq (3) and henceforth.

In this approach, the optimization problem consists of calculating the Lagrangian saddle point (stationary point) using an optimization algorithm. Usually, local optimization techniques are employed because of the large number of variables and the computational cost [6]. Thus, we need to compute not only the objective function but also its gradient. In this way, taking the derivative of $L(m,u_{s},v_{s})$ with respect to model parameters, seismic wavefield and the Lagrange multiplier, we obtain:

$$\begin{array}{c} \frac{\partial L(m,u_{s},v_{s})}{\partial m}={\sum_{s}\left(\frac{\partial A(m)u_{s}}{\partial m}\right)}^{†}v_{s}, \end{array}$$ (4)

$$\begin{array}{c} \frac{\partial L(m,u_{s},v_{s})}{\partial u_{s}}=\sum_{s,r}\Gamma_{s,r}^{†}W_{s,r}^{†}(\Gamma_{s,r}u_{s}-d_{s,r})+\sum_{s}A^{†}\left(m\right)v_{s}, \end{array}$$ (5)

and

$$\begin{array}{c} \frac{\partial L(m,u_{s},v_{s})}{\partial v_{s}}=\sum_{s}A\left(m\right)u_{s}-q_{s}, \end{array}$$ (6)

where the superscript symbol † refers to the adjoint (transpose conjugate).

In the stationary point, note that Eq (6) becomes

$$\begin{array}{c} \frac{\partial L(m,u_{s},v_{s})}{\partial v_{s}}=0\Rightarrow A\left(m\right)u_{s}=q_{s}, \end{array}$$ (7)

which is the constraint in (1). In other words, this constraint is automatically eliminated by solving the wave equation at each iteration. Likewise, from Eq (5):

$$\begin{array}{c} \frac{\partial L(m,u_{s},v_{s})}{\partial u_{s}}=0\Rightarrow A^{†}\left(m\right)v_{s}=-\sum_{r}\Gamma_{s,r}^{†}W_{s,r}^{†}(\Gamma_{s,r}u_{s}-d_{s,r}), \end{array}$$ (8)

obtained the so-called adjoint wave equation [33], in which *v*_*s*_ is computed by back propagating the data residuals.

In summary, the optimization problem formulated in (1) can be rewritten as an unconstrained optimization problem of the form

$$\begin{array}{c} \underset{m}{min}\;\phi \left(m\right):=\frac{1}{2}\sum_{\omega }\sum_{s,r}\|\Gamma_{s,r}u_{s}\left(m,\omega \right)-d_{s,r}\left(\omega \right){\|}_{W_{s,r}}^{2} \end{array}$$ (9)

and the gradient of *ϕ*(*m*) is computed by solving the wave equation,

$$\begin{array}{c} A\left(m,\omega \right)u_{s}\left(\omega \right)=q_{s}\left(\omega \right), \end{array}$$ (10)

and its adjoint equation,

$$\begin{array}{c} A^{†}\left(m,\omega \right)v_{s}\left(\omega \right)=-\sum_{r}\Gamma_{s,r}^{†}W_{s,r}^{†}(\Gamma_{s,r}u_{s}\left(m,\omega \right)-d_{s,r}\left(\omega \right)), \end{array}$$ (11)

In this way, based on Eq (4), the gradient of *ϕ*(*m*) is given by:

$$\begin{array}{c} \nabla_{m}\phi \left(m\right)=\sum_{\omega }\sum_{s}\omega^{2}\;diag{\left(u_{s}(m,\omega )\right)}^{†}v_{s}\left(\omega \right). \end{array}$$ (12)

Note that to compute ∇_*m*_ *ϕ*(*m*) we need to solve numerically two times the wave equation, Eqs (10) and (11). For more details of the adjoint-state method see Ref [33].

### Frequency-dependent offset-preconditioning

A commonly used strategy to reduce the FWI non-linearity is based on a multiscale approach [19, 20]. In this context, the weighting of the objective function is related to a frequency scale, see for instance Ref [34]. A very interesting approach was suggested by Ref [25], in which the data residuals are weighted by the weighting operator, *W*_*s*,*r*_, to balance the relative data contribution. In this approach, the amplitude gain is associated with source-receiver distance (offset) and is applied to each seismic trace. This operator assumes the form:

$$\begin{array}{c} W_{s,r}\left(O_{sr}\right)=exp(g\;ln(|O_{sr}|)), \end{array}$$ (13)

where *O*_*sr*_ is the source-receiver distance (offset) (in meters) and *g* is a scalar that controls the amplitude gain.

In this paper, we propose a modification of weighting operator introduced by Ref [25], Eq (13), in order to control the amplitude according to the frequency and also the offset. In this way, we postulate a *g*-variation according to the expression: *g* = 1/*f*, where *f* = *ω*/2*π* is a magnitude that is included in the scale factor of the weighting, which is a frequency-dependent offset-preconditioning in the inversion process. We postulate this new operator based on the premise that the data components recorded at far offset are more non-linear than the waveforms recorded in the near offset range [28]. Despite this non-linearity problem, the waveforms recorded at far offset are extremely important for recovering the deepest regions of the subsurface models [27]. Thus, in order to reduce the non-linearity of the problem, and therefore, do not discard this far offset information, the waveforms recorded in the largest offsets will have greater weight especially for low frequencies. Considering only the low frequency components of the seismic data, the objective function is more smooth [20], in the sense that it has fewer local minimums than the same objective function calculated for data with high-frequency components.

**Fig 1:**
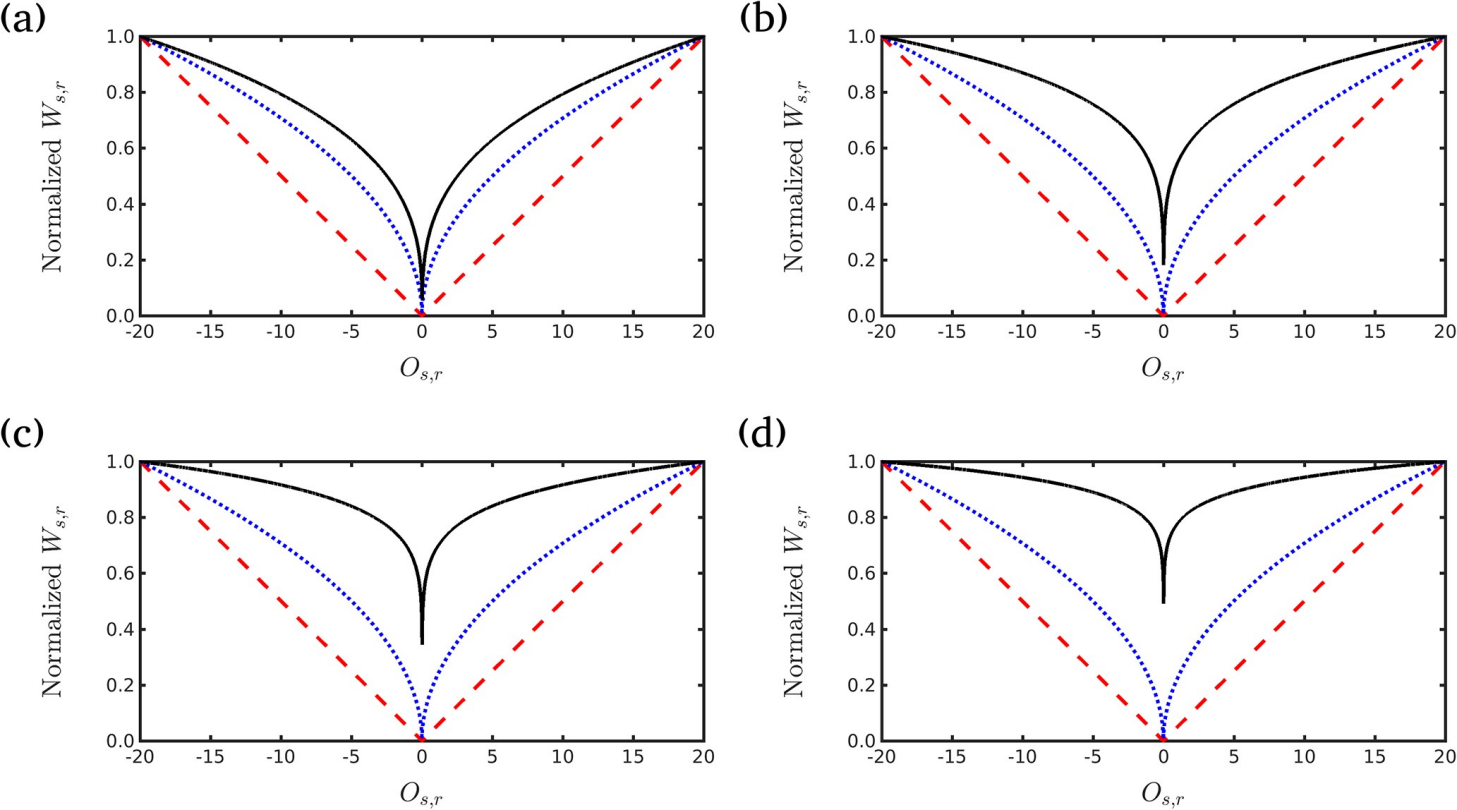
Weighting operator of data misfit. Normalized weighting operator for values of *g* = 1.0, *g* = 0.5 and *g* = 1/*f* (our approach) for the following frequencies: A: *f* = 3*Hz*, B: *f* = 5*Hz*, C: *f* = 8*Hz*, and D: *f* = 12*Hz*.

For comparison, Fig 1 shows weighting operator behavior for frequencies 3, 5, 8, and 12*Hz* considering a maximum offset of 20 km with values of *g* = 1.0 and *g* = 0.5 (as suggested by Ref [25]) and the *g* = 1/*f* case (our proposal). The proposed approach dampens the effect of direct waves on water (especially for short offsets) and reflections close to the receivers and emphasizes the information from the long-offsets, which, in general, carry information from deep geological structures. We notice that for low frequencies, *f* = 3*Hz*, the proposed attenuation is similar to the case *g* = 1, but high frequency, *f* = 12*Hz*, shows a strong attenuation for large offsets.

## Numerical example

**Fig 2:**
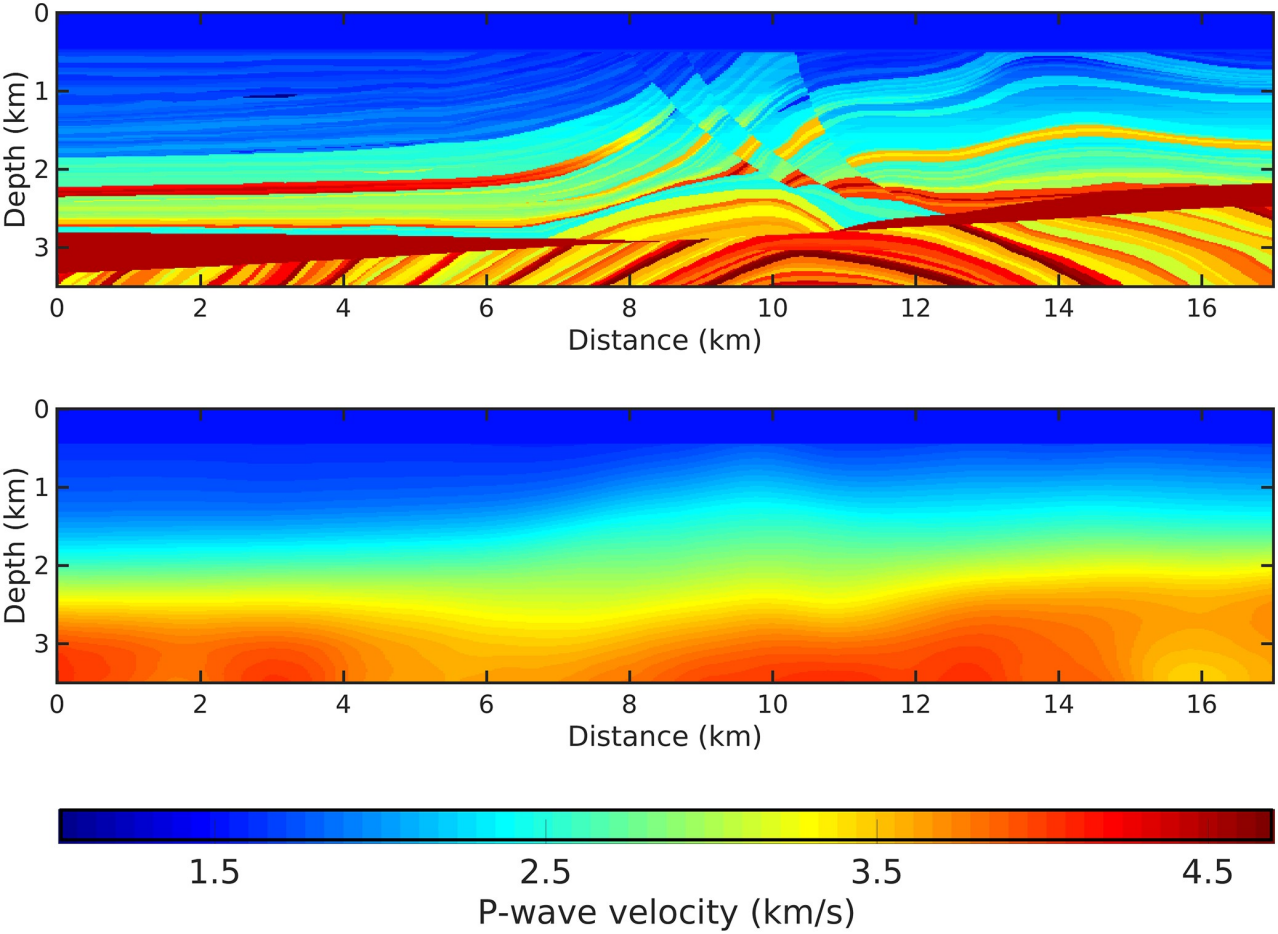
The model for the simulation. A: The Marmousi2 velocity (true model), we employ the P-wave. B: The initial model produced with the true model by using a Gaussian filter with 400m standard deviation.

In order to illustrate the effectiveness of our proposal, we applied the methodology to the Marmousi2 model [29], which is depicted in Fig 2a. This model is based on the geology of the Kwanza Basin region (Angola) and it is 17 km long and a maximum depth of 3.5 km. The P-wave velocities in the model vary from a minimum of 1.03 km/s to a maximum of 4.70 km/s.

**Fig 3:**
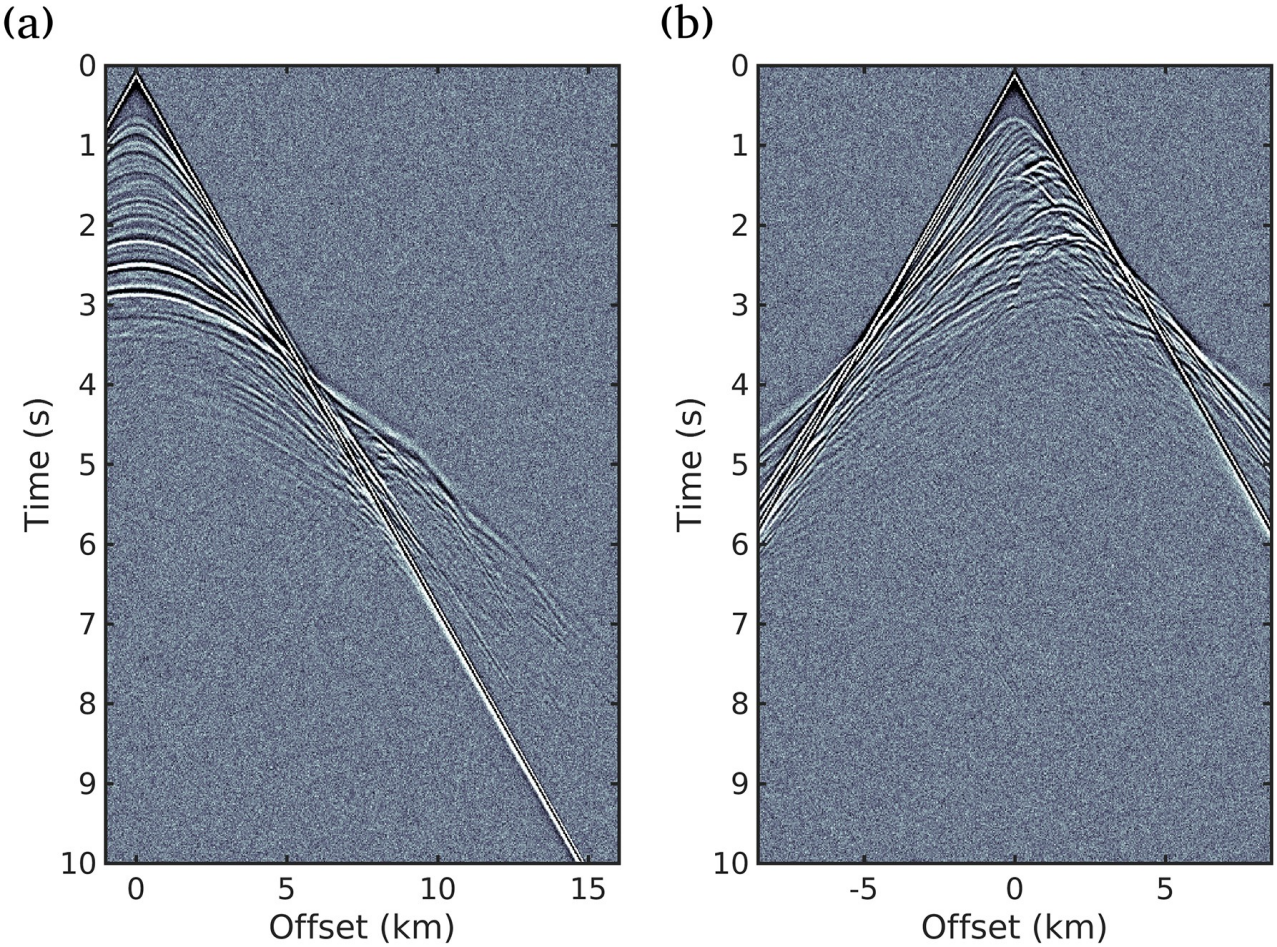
Example of the observed data: Seismograms of the 5*th* (distance 0.9*km*) and 42*nd* (distance 8.3*km*) seismic sources.

We consider an in-line acquisition geometry consisting of 85 sources and 426 receivers. The seismic sources are located every 200 m, from 100 m and 16,900 m. The receivers are located every 40 m. The receivers and the sources are situated at 50 m depth. Moreover, we consider a Ricker wavelet with a peak frequency at 10 Hz as the seismic source [35], and the acquisition time was 10*s*. To avoid the inversion crime, the synthetic observed data set was generated in the time domain with a regular discretization of 5*m* and it was contaminated by Gaussian noise with a signal-to-noise ratio of 15*dB*. Fig 3 shows examples of the observed data (seismograms) for the 5*th* (distance 0.9*km*) and 42*nd* (distance 8.3*km*) seismic sources, respectively.

The employed optimization algorithm was the quasi-Newton method so-called *limited-memory Broyden–Fletcher–Goldfarb–Shanno* (*l*-BFGS) [36]. The *l*-BFGS is based on the iterative updating of the model parameters (*m*) according to:

$$\begin{array}{c} m_{j+1}=m_{j}-\alpha_{j}H^{-1}\nabla_{m}\phi \left(m_{j}\right) \end{array}$$ (14)

where *α*_*j*_ is the step-length that is computed through a line search procedure that satisfies the Wolfe conditions [36]. In addition, ∇_*m*_ *ϕ*(*m*_*j*_) = ∂*ϕ*(*m*_*j*_)/∂*m* is given by the gradient of the objective function *ϕ*(*m*_*j*_). We note that, the gradient is computed using the model parameters at the *j*-th iteration, given by Eq (12) and **H**^−1^ is the *l*-BFGS approximation to the objective-function Hessian inverse.

To start the inversion, we use the initial model shown in Fig 2b. This initial model was obtained by smoothing the true model using a Gaussian filter with 400*m* of standard deviation. For sake of comparison, we consider three different scenarios: (i) In the first one, we consider only waveforms recorded at a maximum offset of 4 km, while in the second scenario (ii) the maximum offset considered was 8 km; (iii) In the last one, all receivers are considered (all-offsets). For each scenario, we perform four numerical experiments, in which different *g*-values are employed to build the weighting operator, Eq (13): *g* = 0 (Conventional FWI); *g* = 1/*f* (proposed in this study); *g* = 1 and *g* = 0.5 (the last two ones as suggested by Ref [25]). The multiscale FWI process has been performed in the frequency domain, with four frequency groups: {3,4,5}, {5,6,7}, {8,9,10} and {11,12,13} Hz. Moreover, we do not consider a maximum number of *l*-BFGS iterations. The stop criteria for the optimization process was the tolerance, *ϵ*, in the gradient norm: ||∇_*m*_ *ϕ*(**m**)|| < *ϵ* = 10^−10^, which imposes a limit for the velocity model updating.

**Fig 4:**
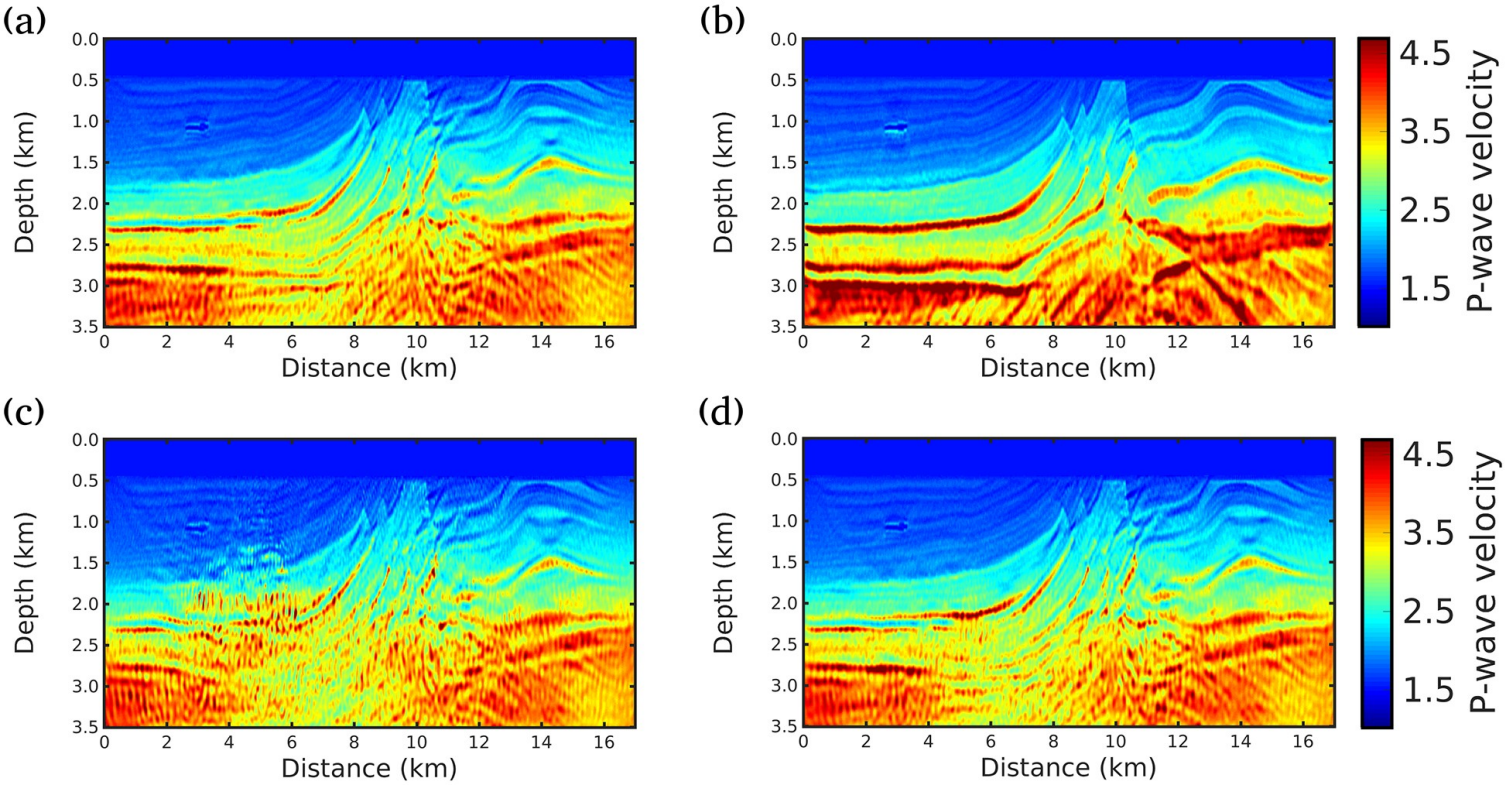
FWI results for scenario (i) (Maximum offset of 4km). Acoustic velocity model models built by A: conventional FWI, B: g = 1/f (our proposal), C: g = 1.0, and D: g = 0.5.

**Fig 5:**
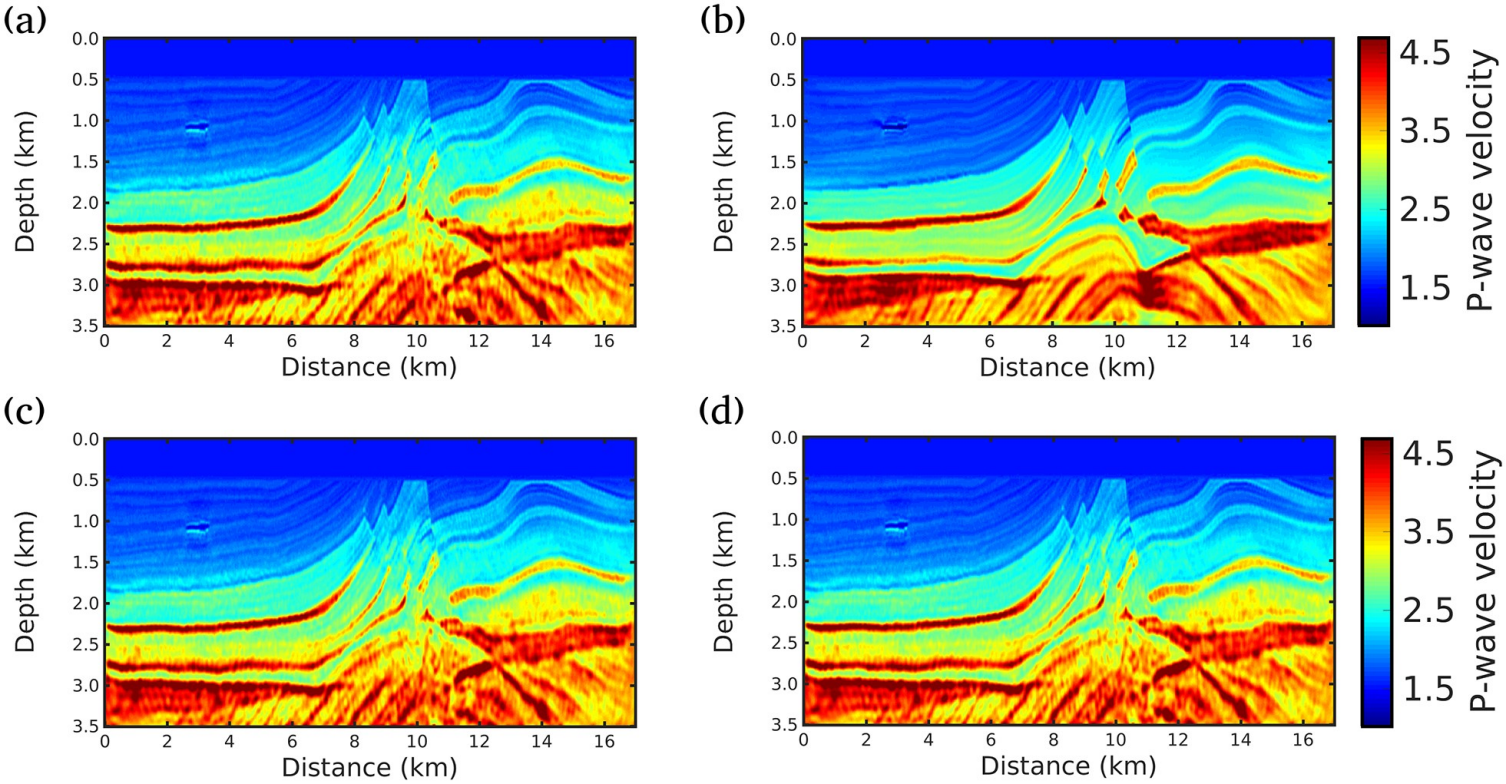
FWI results for scenario (ii) (Maximum offset of 8km). Acoustic velocity model models built by A: conventional FWI, B: g = 1/f (our proposal), C: g = 1.0, and D: g = 0.5.

**Fig 6:**
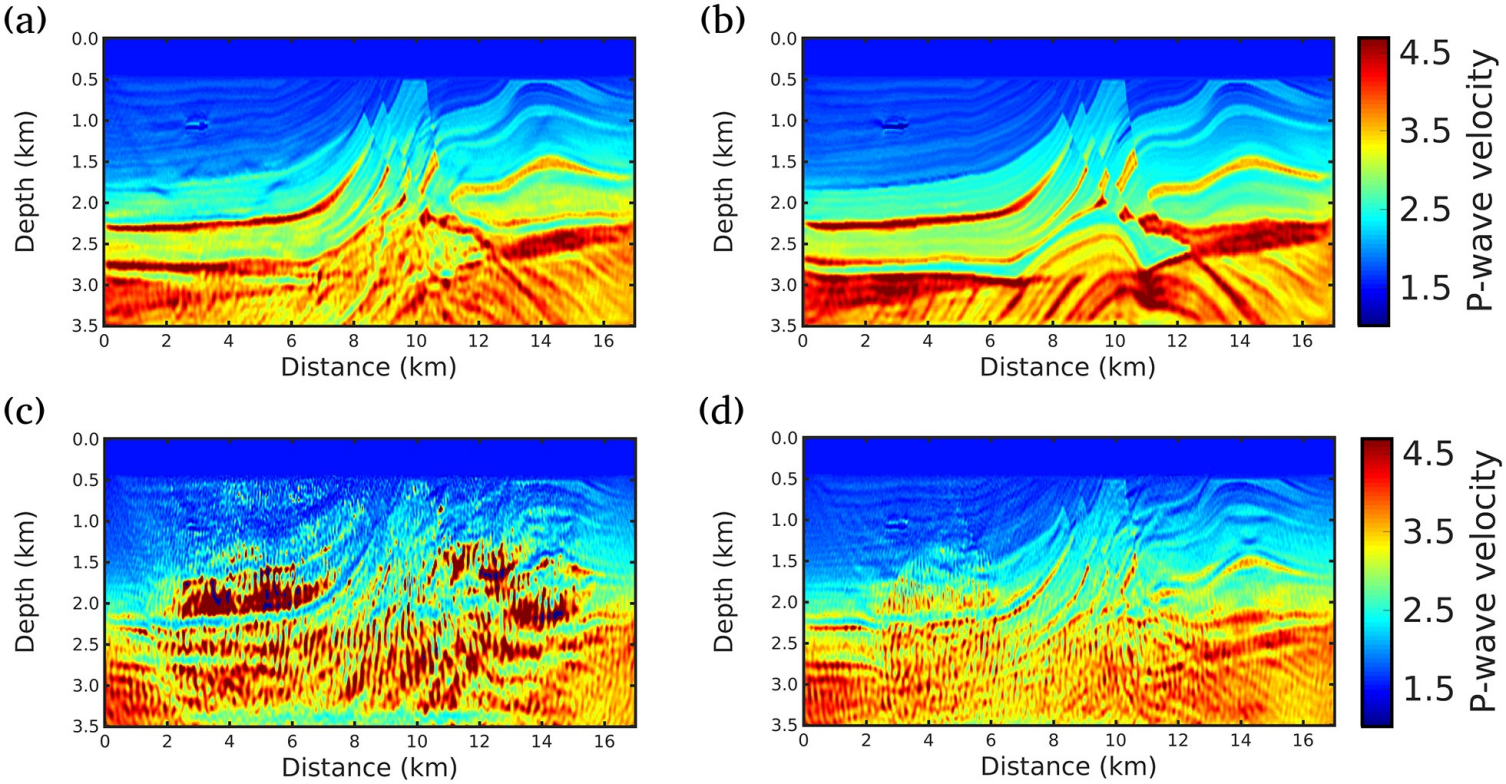
FWI results for scenario (iii) (all receivers). Acoustic velocity model models built by A: conventional FWI, B: g = 1/f (our proposal), C: g = 1.0, and D: g = 0.5.

Figs 4, 5 and 6 show the inversion results for the three scenarios considered in this study. As can be seen in Fig 4 (scenario (i)), all the strategies presented in this study fail to reconstruct the deeper structures of the subsurface, especially for depths greater than 2*km*. This is due to the fact that only the near field is taken into account, that is, low or almost no importance is given to the diving waves (see Fig 3 for |Offset| > 4*km* and Time> 4*s*), that carry information from the deeper geological structures. This scenario was included to show the importance of large offsets in seismic imaging.

When an acquisition with a wider-angular aperture is considered in scenario (ii), the inversion results outperform the FWI results of the scenario (i), as depicted in Fig 5. We notice that the velocity model obtained by our proposal is the closest to the true model. Note, for instance, the region located at 1.08 km in depth and 3 km in the lateral location and the region below this area; In this region, our proposal was able to achieve a more accurate image of the subsurface without the artifacts produced by the other approaches, Fig 5a, 5C and 5d. Indeed, the presence of a gas-charged sand channel, with 38 m of thickness and low-velocity (1.03 km/s), makes the reconstruction challenging in this region, because it generates a strong diffraction point causing interference on the wavefront. However, our proposal was almost immune to these difficulties. The artifacts caused by the gas-charged sand channel are better damped by our proposal. It should also be noted that the best resolution achieved by our proposal in the region below 3 km and between the distances 7 and 11 km, where water-wet sand, gas and oil cap hyperbolic layers occurs.

Finally, in scenario (iii), in which all receivers are considered, the results for *g* = 1.0 and *g* = 0.5 are worse than the other scenarios for all strategies. This shows that the seismic data recorded in far field must be used carefully, as suggested by Ref [28]. Again, the proposed strategy proved to be stable and outperforms other strategies, as depicted in Fig 6b.

Since the true model is known in this study, we compared it with the FWI results for the four strategies presented above. We use four statistical measures: the *L*_1_ − *norm*, the Normalized Root Mean Square (NRMS), the Pearson correlation coefficient R [37], and the Structural Similarity Index (SSIM) [38]. For the first two measures we use the residuals (*c*^(*true*)^—*c*^(*inv*)^) where *c*^(*true*)^ represents the velocities of the true model and *c*^(*inv*)^ the velocities of the inverted model (the reconstructed P-wave velocity model). The second measure is the NRMS of the residuals which is defined as:

$$\begin{array}{c} \text{NRMS}={\left[\frac{{\sum_{i}\left(c_{i}^{\left(true\right)}-c_{i}^{\left(inv\right)}\right)}^{2}}{{\sum_{i}\left(c_{i}^{\left(true\right)}\right)}^{2}}\right]}^{1/2}, \end{array}$$ (15)

where the NRMS varies from 0 (perfect fit) to ∞ (bad fit). The last two measures are similarity metrics between images: one is the SSIM index, and the other is the correlation (R). These similarity measures vary from −1 (bad similarity) to 1 (perfect similarity). Table 1 shows a comparison of the results from the *L*_1_-norm error, NRMS, SSIM, and R.

**Table 1:** Main statistics of the numerical example: The *L*_1_-norm and the Normalized Root Mean Square (NRMS) statistics are based on the misfit between the true model and the model inverted according to the explored methodologies (*c*^(*true*)^—*c*^(*inv*)^). The Pearson correlation coefficient (R) and the Structural Similarity Index (SSIM) measure similarities between images.

Strategy	Scenario	*L*_1_-norm	NRMS	R	SSIM
Conventional FWI	(i)	101.92	34.23	.8804	.3790
	(ii)	81.69	20.65	.8976	.4777
	(iii)	98.50	23.30	.8952	.4955
Our proposal (*g* = 1/*f*)	(i)	105.80	20.50	.8996	.4844
	(ii)	98.85	15.15	.9674	.7195
	(iii)	95.97	18.95	.9696	.7226
Ref [25] (*g* = 1.0)	(i)	104.88	39.99	.8712	.3540
	(ii)	105.51	22.79	.9005	.4793
	(iii)	204.66	164.91	.7038	.2653
Ref [25] (*g* = 0.5)	(i)	101.02	37.39	.8806	.3766
	(ii)	105.07	23.56	.8998	.4821
	(iii)	109.27	35.62	.8671	.3511

We notice in Table 1 that the image corresponding to our proposed methodology has the lowest NRMS and *L*_1_-norm misfit, which means the lowest pixel-by-pixel error. In addition, our image also shows the highest R and SSIM, indicating the highest similarity with the true model than the other approaches.

**Fig 7:**
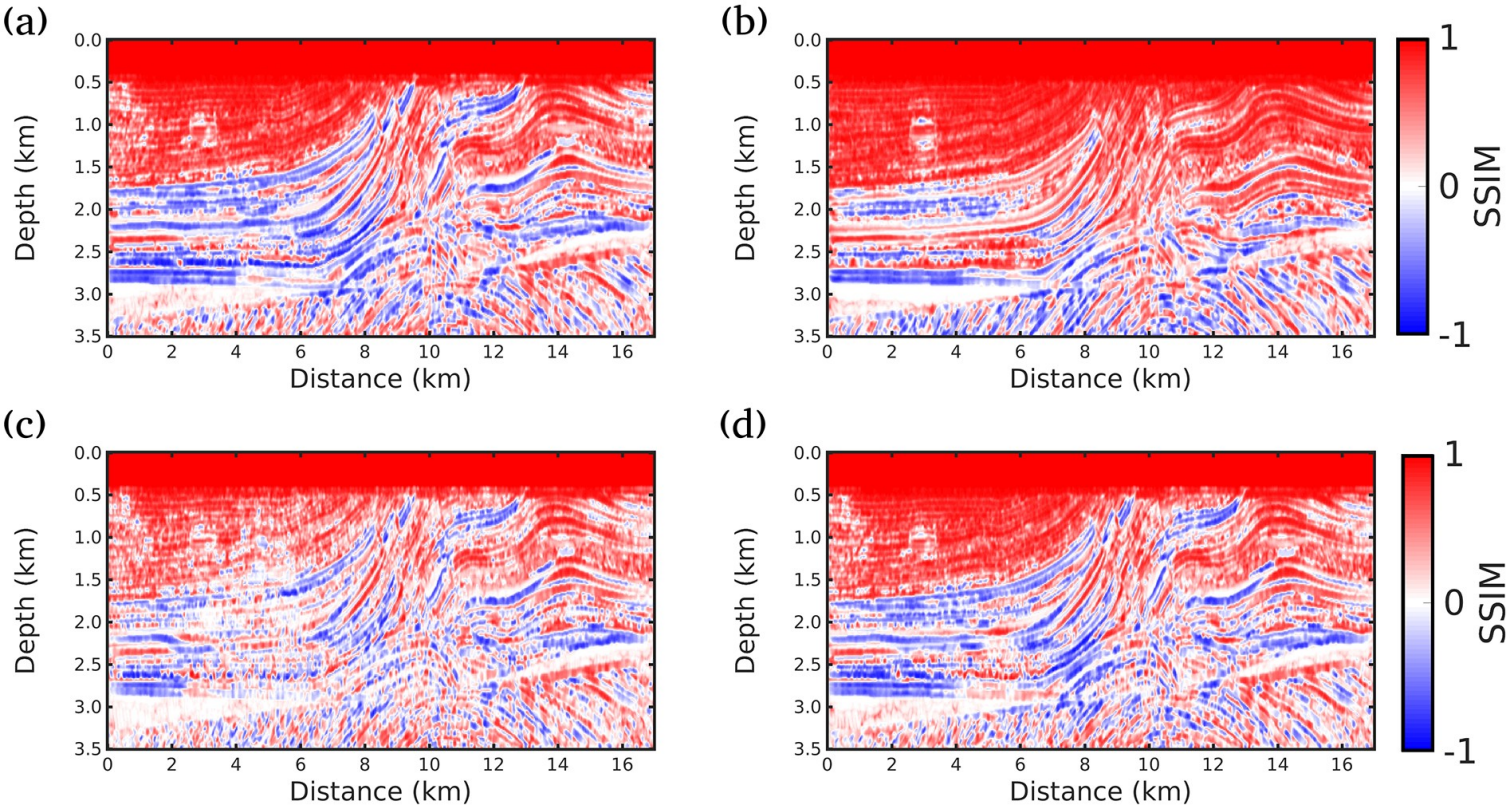
Similarity analysis for scenario (i) (Maximum offset of 4km). SSIM maps between the true model and inverted models by A: Conventional FWI (*g* = 0); B: *g* = 1/*f* (our proposal); C: *g* = 1.0 and D: *g* = 0.5. Red color indicates the highest similarity.

**Fig 8:**
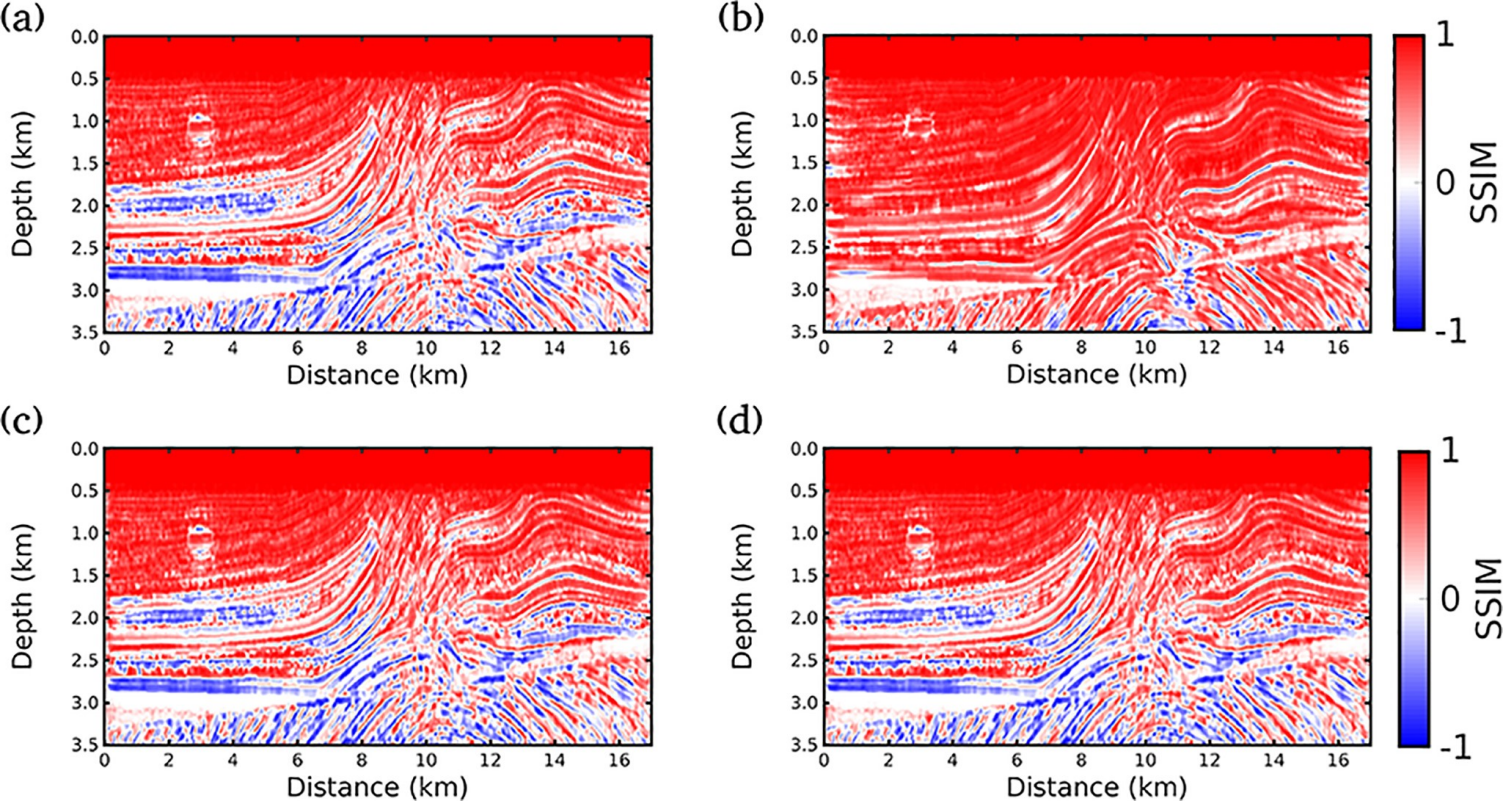
Similarity analysis for scenario (ii) (Maximum offset of 8km). SSIM maps between the true model and inverted models by A: Conventional FWI (*g* = 0); B: *g* = 1/*f* (our proposal); C: *g* = 1.0 and D: *g* = 0.5. Red color indicates the highest similarity.

**Fig 9:**
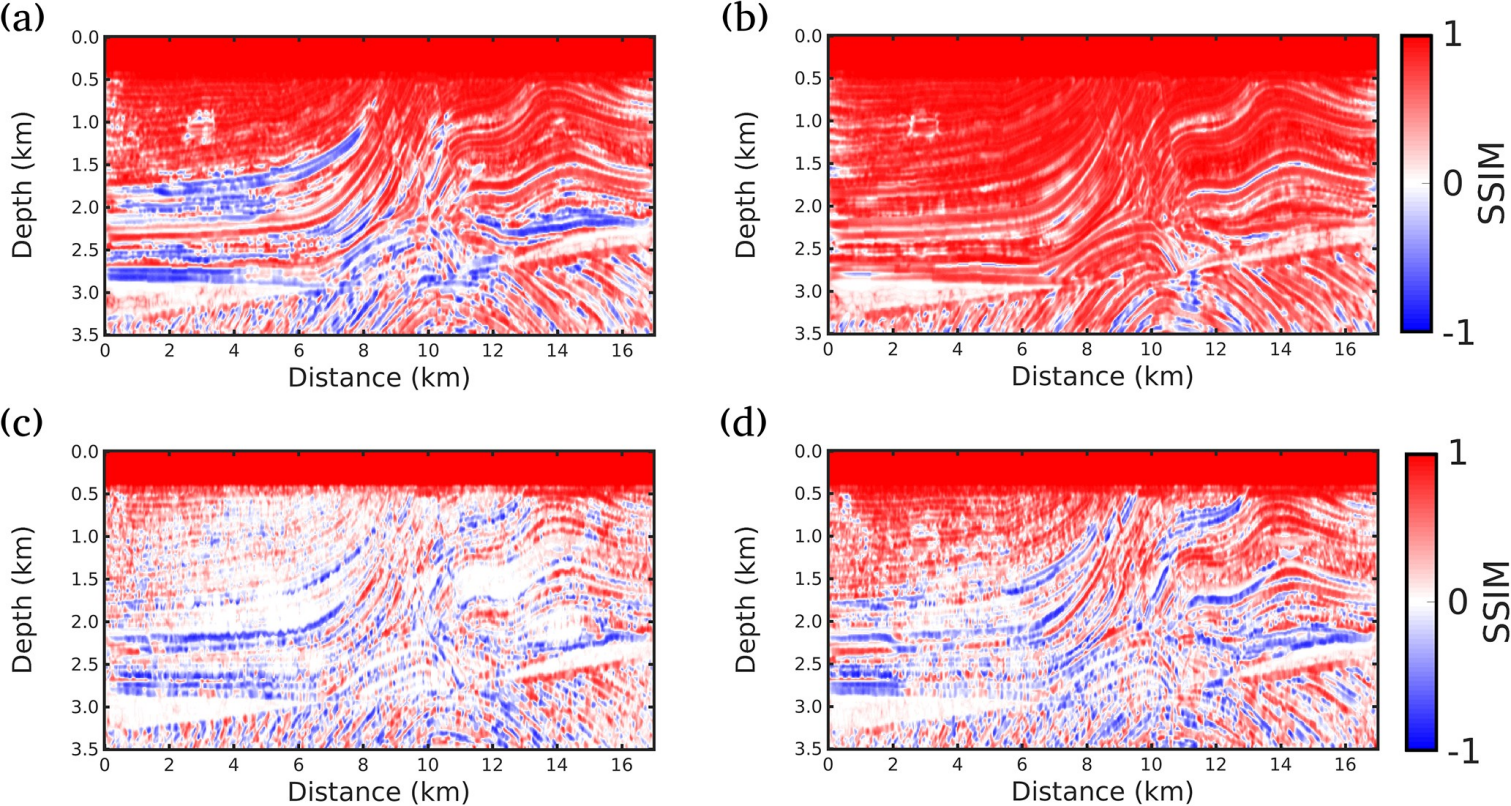
Similarity analysis for scenario (iii) (all receivers). SSIM maps between the true model and inverted models by A: Conventional FWI (*g* = 0); B: *g* = 1/*f* (our proposal); C: *g* = 1.0 and D: *g* = 0.5. Red color indicates the highest similarity.

Finally, Figs 7, 8 and 9 show the local SSIM maps between the true model and inverted models by the Conventional FWI (*g* = 0), *g* = 1/*f* (our proposal), *g* = 1.0 and *g* = 0.5, for scenarios (i), (ii) and (iii), respectively. These figures indicate that our proposal produces the best velocity model correlation with the true model (the largest area in red). Here, to estimate the local SSIM for each pixel we employ a sample centered on the pixel weighted with an isotropic Gaussian function with standard deviation 1.5.

## Conclusion

In this paper, we have presented an efficient strategy to mitigate the FWI non-linearity for wide-aperture seismic data-inversion. Based on the data-offset-preconditioning for FWI introduced by Ref [25], we proposed an improvement for the weighting of information, which is based on a frequency-data-offset-preconditioning. The numerical results show that our strategy outperforms other approaches examined in this study, especially when seismic data recorded in long offsets are taken into account. Our methodology emphasizes information from deep structures for long source-receiver distance and dampens the information of short distances when high frequencies are inverted.

Our experiments were performed using a compute hosting a Quad-core (Intel Xeon E5-1620 v3) processor at 3.50GHz and 256 GB RAM, in which each *l*-BFGS iteration takes about 111.93 ± 0.47 seconds regardless of the strategy adopted. In other words, our proposal provides better seismic reconstruction without additional computational cost compared to the traditional approach. Finally, we think that the FWI based on the operator presented in this study is a promising alternative for imaging wide-aperture seismic data. As a perspective, we intend to investigate the performance of our proposal in the viscoelastic FWI case, in which wave-energy dissipation is considered. We believe that the strategy proposed in this work is able to properly balance the waveform amplitudes for a wide range of offsets.
